# The Significance of Scalp Involvement in Pemphigus: A Literature Review

**DOI:** 10.1155/2018/6154397

**Published:** 2018-03-25

**Authors:** Marta Sar-Pomian, Lidia Rudnicka, Malgorzata Olszewska

**Affiliations:** Department of Dermatology, Medical University of Warsaw, Warsaw, Poland

## Abstract

Scalp is a unique location for pemphigus because of the abundance of desmogleins localized in hair follicles. Scalp involvement is observed in up to 60% of patients in the course of pemphigus. The lesions may occasionally lead to alopecia. Unforced removal of anagen hairs in a pull test is a sign of high disease activity. Direct immunofluorescence of plucked hair bulbs is considered a reliable diagnostic method in patients with pemphigus. Follicular acantholysis is a characteristic histopathological feature of pemphigus lesions localized on the scalp. Trichoscopy may serve as a supplementary method in the diagnosis of pemphigus. This review summarizes the most recent data concerning scalp involvement in pemphigus vulgaris and pemphigus foliaceus. A systematic literature search was conducted in three medical databases: PubMed, Embase, and Web of Science. The analysis included literature data about desmoglein distribution in hair follicles, as well as information about clinical manifestations, histopathology, immunopathology, and trichoscopy of scalp lesions in pemphigus and their response to treatment.

## 1. Introduction

Pemphigus is a group of autoimmune bullous diseases, in which anti-desmoglein autoantibodies induce the loss of keratinocyte adhesion, leading to blister formation. Two main types of pemphigus may be distinguished: pemphigus vulgaris (PV) and pemphigus foliaceus (PF). Desmoglein 3 is considered to be the main autoantigen in pemphigus vulgaris. Additionally, anti-desmoglein 1 autoantibodies may be detected in more than half of patients with pemphigus vulgaris [1]. Desmoglein 1 is the main autoantigen of pemphigus foliaceus. Mucosal lesions with or without skin lesions are observed in pemphigus vulgaris, whereas in pemphigus foliaceus only cutaneous involvement is observed. The diagnosis of pemphigus is based on the clinical picture, direct and indirect immunofluorescence, and histopathology as well as anti-desmoglein 1 and 3 enzyme-linked immunoassay.

The frequency of scalp involvement in the course of pemphigus is estimated at 16–60% [2–4]. According to literature data, the scalp is the first location in 9–15% of patients with pemphigus [3, 4]. Several cases of alopecia in the course of pemphigus have been described [5]. The significance of the distribution of desmogleins in hair follicles for scalp involvement [6] and a potential use of direct immunofluorescence of plucked hairs are discussed in the literature [7]. The significance of scalp involvement for the course of pemphigus remains controversial.

The aim of this systematic review was to collect and critically analyze data concerning the types of alopecia occurring in pemphigus, the usability of direct immunofluorescence of plucked hairs as well as trichoscopy in diagnosing pemphigus, the histopathological picture of scalp involvement in pemphigus, and the responsiveness to treatment in patients with scalp involvement in the course of pemphigus.

## 2. Material and Methods

**Figure 1:**
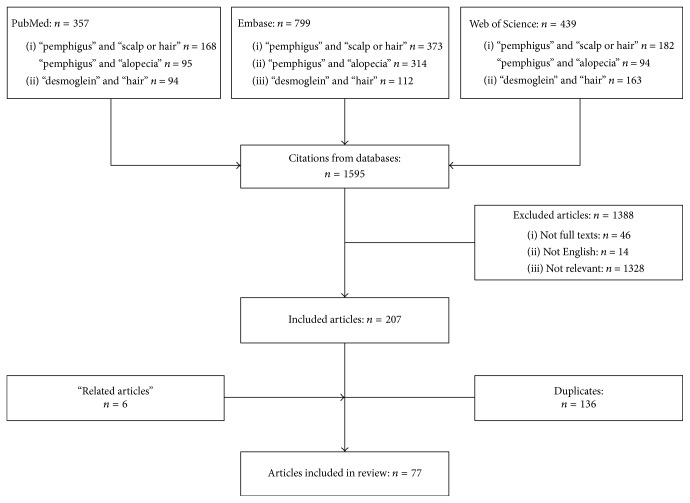
Flow chart.

A systematic review of the literature was conducted in three electronic medical databases: PubMed, Embase, and Web of Science (till February 10, 2018). The search with the conjunction of search terms “pemphigus” AND “scalp OR hair”, “pemphigus” AND “alopecia”, “desmoglein” AND “hair” returned a total number of 1595 articles. Only studies conducted in humans and English-language articles were included. Non-full-text publications, duplicates, and nonrelevant articles were excluded. Publications on endemic pemphigus foliaceus and paraneoplastic pemphigus were excluded. Eventually, 77 manuscripts found in the databases were included (Figure 1). We reviewed published data related to desmoglein distribution in hair follicles, as well as the clinical manifestations, histopathological, immunopathological, and trichoscopic features of scalp involvement in pemphigus. The response of scalp lesions to treatment was analyzed.

## 3. Results and Discussion

### 3.1. Desmoglein Expression in Hair Follicles

Desmogleins are the main autoantigens of pemphigus. Desmogleins (Dsgs) are calcium-dependent glycoproteins belonging to the desmosomal cadherin superfamily. Four types of desmogleins have been described. Desmoglein 1 (Dsg1) is expressed in the inner root sheath and the innermost layers of the outer root sheath, as well as epidermal suprabasal cells [6]. Hypotrichosis with microscopically normal hairs was observed in a mutation of Dsg1 gene causing SAM syndrome (severe dermatitis, multiple allergies, and metabolic wasting) [8]. Desmoglein 2 (Dsg2), an antigen of a less prominent role in pemphigus, is highly expressed in the least differentiated cells of the cutaneous epithelium, including the hair follicle bulge of the fetus and adult, bulb matrix cells, and the basal layer of the outer root sheath (ORS) [6]. The expression of desmoglein 3 in the hair follicle depends on the keratinization type. In the infundibulum of the hair follicle keratinization resembles the one in the epidermis (epidermal keratinization), whereas in the isthmus the granular layer is absent (trichilemmal keratinization) [6]. In the areas of trichilemmal keratinization of the hair follicle desmoglein 3 is expressed throughout all layers of the ORS, while in the areas of epidermal keratinization its expression is limited mainly to the basal layer. Moreover, there is a strong expression of Dsg3 detected in the medulla [6]. Desmoglein 4 (Dsg4) is mainly expressed in the hair shaft cortex, the lower hair cuticle, and the upper inner root sheath [9, 10]. Some authors suggest that desmoglein 4 may play a role as an additional autoantigen in pemphigus vulgaris [9]. Mutations in Dsg4 gene are responsible for localized autosomal recessive hypotrichosis [11], monilethrix [12], and in some cases an overlap of both [13]. Schaffer et al. reported three cases of localized autosomal recessive hypotrichosis and monilethrix in combination with scalp erosions presenting mutations in the gene for desmoglein 4 [14].

### 3.2. Clinical Manifestations of Scalp Involvement in Pemphigus

**Figure 2:**
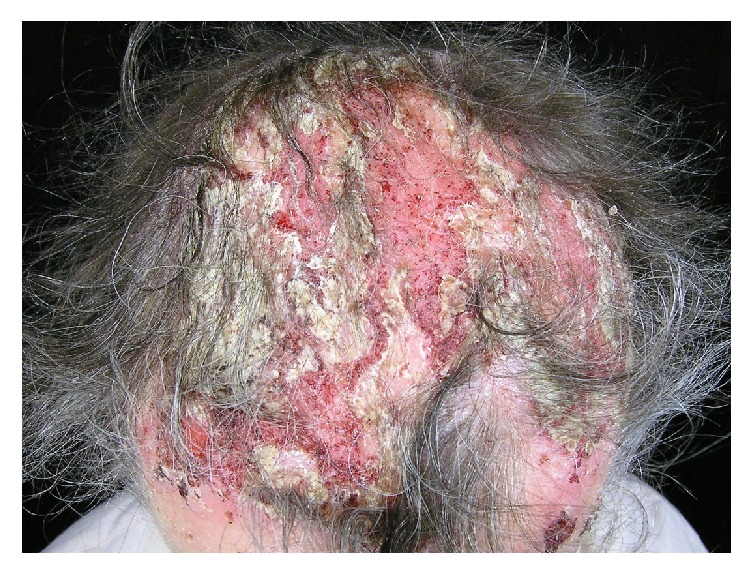
Scalp involvement in a patient with pemphigus vulgaris.

**Figure 3:**
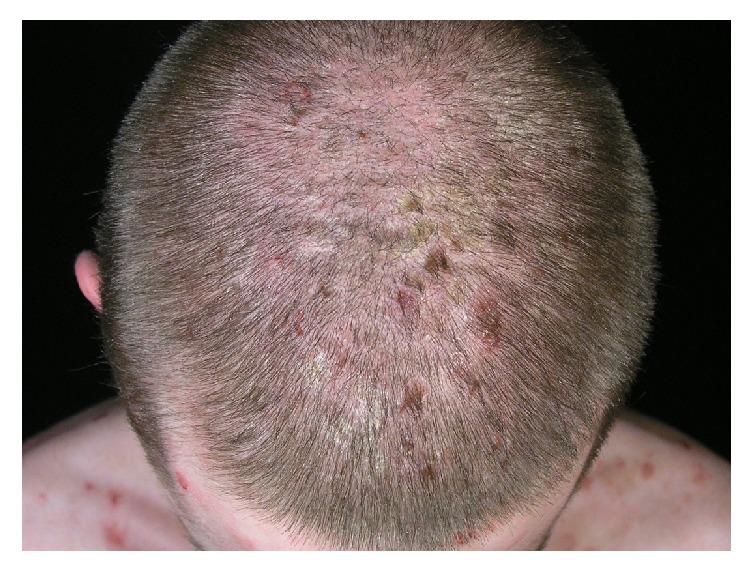
Scalp involvement in a patient with pemphigus foliaceus.

In the course of pemphigus vulgaris and pemphigus foliaceus scalp lesions manifest as erosions and crusted or scaly plaques (Figures 2 and 3). Cases of pemphigus vegetans, a variant of pemphigus vulgaris, with lesions localized on the scalp were also described [15–18]. In five cases scalp lesions constituted an isolated manifestation of pemphigus vegetans [15–18]. Unilesional pemphigus vulgaris on the scalp was also described in two cases. In both patients the development of the lesions followed a surgical intervention, in one of the patients a cochlear implantation [19] and a hair transplant in the other [20]. It was hypothesized that trauma may facilitate the overpresentation of epidermal antigens by disturbing the dermoepidermal junction [21]. Scalp erosions limited to the scalp were also reported in the course of pemphigus foliaceus [22, 23]. According to our literature search, no cases of pemphigus erythematosus limited to the scalp were reported to date.

*Hair Loss in Pemphigus.* Scalp lesions in pemphigus may lead to alopecia. Nonscarring alopecia was reported to be an unusual manifestation of scalp pemphigus [24]. Veraitch et al. described 5 patients with pemphigus vulgaris and progressive hair loss with alopecic patches [5]. In this study, apart from characteristic histopathological features of pemphigus, bacterial culture revealed the presence of* Staphylococcus epidermidis*,* Staphylococcus aureus*, and* Klebsiella pneumoniae*. The authors suggested that the combination of anti-desmoglein autoantibody-mediated acantholysis with a concomitant infection may cause the weakening of the hair follicle anchorage and result in alopecia [5]. Hadayer et al. reported a case of a patient with pemphigus vulgaris, in whom alopecia was also accompanied by* Staphylococcus aureus* infection [24]. Our literature search revealed only one case report of nonscarring alopecia occurring in pemphigus foliaceus [25]. In this publication, the widespread erosions and alopecia were the first manifestations of the disease in a 14-year-old girl. The authors suggested that the occurrence of alopecia in this case could have been associated with an extremely high anti-Dsg1 antibody concentration, equaling 2017 U/ml (cut off value < 14 U/ml). Recently, Yoshida et al. described a case of transition from pemphigus foliaceus to pemphigus vulgaris [26]. The authors observed a correlation between scalp lesions and anti-Dsg3 antibody concentration in enzyme-linked immunoassay [26].

To date, scarring alopecia has been described in 7 patients with pemphigus vulgaris [27–32]. Five of them developed tufted hair folliculitis in the course of prolonged scalp involvement [28–32]. In 5 cases bacterial culture was positive, showing the presence of* Staphylococcus aureus *[30–33] or* Staphylococcus epidermidis* [28]. It is unclear whether bacteria may act as an additional factor leading to alopecia. Gaitanis et al. [27] and Lapiere et al. [33] described cases of pemphigus vulgaris in which scarring alopecia was not accompanied by tufting. In both cases the development of cicatricial alopecia was associated with long-lasting and extensive scalp lesions.

Delmonte et al. first observed that in 3 patients with pemphigus vulgaris anagen hairs with intact root sheaths could be easily removed from both lesional and perilesional scalp by a gentle pull test [34]. The authors of this case report called that phenomenon “normal anagen effluvium.” According to Delmonte et al., the loss of anagen hairs with preserved root sheaths is a manifestation of the subclinical involvement of the hair follicle and may be regarded as a hair equivalent to Nikolsky's sign of the skin [34]. According to Daneshpazhooh et al. anagen hair loss is a common feature of active pemphigus vulgaris. In this article, intact anagen hairs covered by root sheaths were obtained by gentle pull test in 59 of 96 (61.5%) patients with pemphigus vulgaris [35]. Lesional anagen hair loss was observed in 51 of 57 (89.5%) patients, whereas nonlesional anagen hair loss was present in 27 of 57 (47.4%) patients. Mean anagen hair count was significantly higher in severe cases (6.83 +/− 7.89 hairs) as compared to patients with pemphigus vulgaris with a moderate course of the disease (1.06 +/− 1.94 hairs). The authors of this study reported that anagen hair loss was an independent predictor of pemphigus vulgaris severity [35]. In a recent study of Fard et al. performed in 52 patients with newly diagnosed pemphigus vulgaris, a significant correlation between anagen hair loss and the concentration of anti-desmoglein 1 antibody concentration as well as scalp and skin Pemphigus Disease Area Index scores was observed [36].

### 3.3. Measurement Methods for Scalp Involvement in Pemphigus

Scalp lesions in pemphigus may differ in size, number, and activity. Objective measurement tools are needed to evaluate scalp involvement in patients with pemphigus. As regards the most commonly used validated measurement methods evaluating pemphigus disease activity, scalp involvement is separately assessed only with Pemphigus Disease Area Index (PDAI) [37]. The following factors are taken into consideration in scalp PDAI: the distribution of the lesions in scalp quadrants and the size of the lesions as well as their quality, such as the presence of blisters, erosions, or new erythema for active lesions and postinflammatory hyperpigmentation or erythema after resolving lesions (Table 1). In Autoimmune Bullous Skin Disorder Intensity Score (ABSIS) the extent of skin lesions is assessed with the “rule of nine,” where the head and neck area represents 9% of the body surface area. The percentage of body surface area is multiplied by the weighting factor, depending on the activity of the lesions (factor 1.5 for bullae and erosions, factor 1.0 for dry erosions, and factor 0.5 for reepithelized erosions) [38].

In Pemphigus Vulgaris Activity Score (PVAS) only the presence or absence of scalp involvement is evaluated, whereas the quality and number of lesions as well as the presence of Nikolsky's sign are not evaluated separately for the scalp [39].

According to Rahbar et al. PDAI may be recommended for use in multicenter studies because it has the highest interrater reliability as well as the highest convergent validity as compared to ABSIS and PVAS [37].

### 3.4. Histopathological Features of Scalp Involvement in Pemphigus

**Figure 4:**
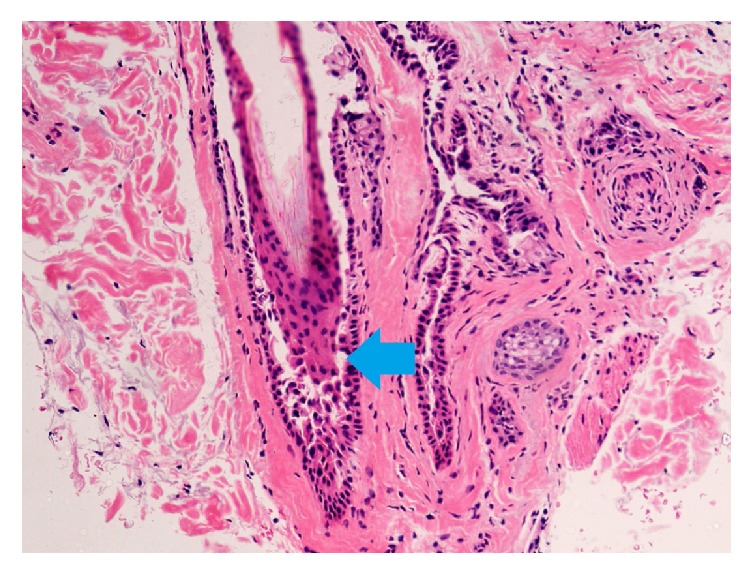
Histopathology of a scalp lesion in pemphigus vulgaris (acantholysis in the outer root sheath of the hair follicle, blue arrow). Hematoxylin and eosin, ×40.

Intraepidermal blister formation due to acantholysis is a characteristic histopathological finding of pemphigus. Acantholysis is located over the basal layer in pemphigus vulgaris and forms the so-called “tombstone” pattern on the floor of the blister [19]. In pemphigus foliaceus, acantholysis is located under the corneal layer [25]. Acantholysis may also be observed in the structures of hair follicles (Figure 4) [24, 27, 40, 41]. Veraitch et al. observed acantholysis between keratinocytes of the outer root sheath extending from the infundibulum to the suprabulbar level in anagen hair follicles of pemphigus vulgaris lesions [5]. A case report of pemphigus foliaceus by Mlynek et al. described acantholysis which was observed in the infundibular outer root sheath, sparing its infrainfundibular part [25]. In our recent study performed on 32 patients with pemphigus, we observed that follicular acantholysis is a common feature of both types of pemphigus occurring in 12/17 (70.6%) patients with pemphigus vulgaris and 12/15 (80.0%) patients with pemphigus foliaceus [42]. We also confirmed that pemphigus vulgaris and pemphigus foliaceus differ as regards the depth of acantholysis. Acantholysis extended down the entire length of the outer root sheath of the hair follicle in pemphigus vulgaris, whereas in pemphigus foliaceus acantholysis was restricted to the infundibulum [42]. Acantholysis was also observed in vellus hair follicles in 12/17 (70.6%) patients with pemphigus vulgaris and 9/15 (60.0%) patients with pemphigus foliaceus [42]. Furthermore, acantholysis was present in sebaceous gland ducts in 3/17 (17.6%) patients with pemphigus vulgaris and 1/15 (6.7%) patients with pemphigus foliaceus as well as sweat gland ducts in 2/17 (11.8%) and 2/15 (13.3%) patients, respectively [42]. We also observed the miniaturization of sebaceous glands, which was seen in 14/17 (82.4%) patients with pemphigus vulgaris and 12/15 (80%) patients with pemphigus foliaceus. Whether this feature is specific for pemphigus or is associated with other comorbidities, for example, androgenetic alopecia, requires further evaluation [42].

Perifollicular infiltrates were observed in long-standing scalp lesions of patients with pemphigus vulgaris [28]. Superficial inflammatory infiltrates consisting of lymphocytes and neutrophils were observed by Veraitch et al. who reported 5 patients with pemphigus vulgaris and progressive hair loss [5]. Interestingly, the infiltrates did not reach the lowermost bulbar region of the hair follicle. Moreover, hair follicle density and hair cycle were unaffected [5].

As regards the course of pemphigus, perivascular inflammatory infiltrates in scalp lesions commonly consisted of lymphocytes [28, 30]; in rare cases they were accompanied by eosinophils [15, 33]. In our study neutrophils were the most common inflammatory cells observed in the epidermis, occurring in about 40% of patients with pemphigus vulgaris and 60% of patients with pemphigus foliaceus [42]. Mononuclear inflammatory infiltrates were predominant in the dermis, occurring in 82% of patients with pemphigus vulgaris and 93% of patients with pemphigus foliaceus [42]. The presence of keratinization disorders was another common histopathological finding observed in scalp pemphigus [42]. Parakeratosis was observed in pemphigus foliaceus [22]. In our study the hyperkeratinization of follicular ostia was observed in 53% of PV patients and 60% of PF patients [42].

### 3.5. Direct Immunofluorescence (DIF) of Plucked Hair

**Figure 5:**
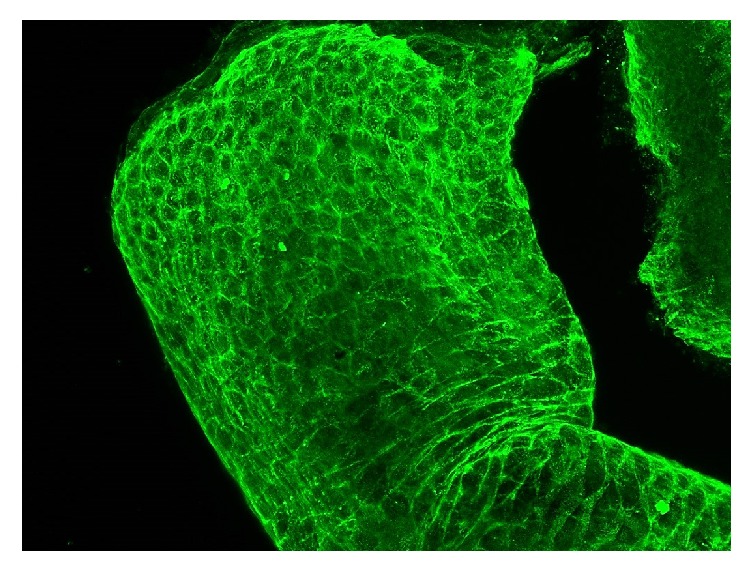
Direct immunofluorescence of a plucked hair showing IgG deposits in the intercellular spaces of the outer root sheath under confocal microscope (LSM70; Zeiss, Jena, Germany, original magnification ×63).

The direct immunofluorescence of plucked hairs may be helpful in the diagnosis of pemphigus (Figure 5). The hairs for direct immunofluorescence are obtained by plucking with forceps [7]. Daneshpazhooh et al. reported that the sensitivity of the direct immunofluorescence of plucked hair equals 91% (100 of 110 patients with PV) [43]. Positive intercellular staining of the outer root sheath of anagen hairs was also reported by Rao et. al in 85% (17/20) of patients with active pemphigus vulgaris [44]. In this study the direct immunofluorescence of plucked hairs was positive in all patients who had scalp lesions and in 77.7% of patients with no scalp lesions in the course of pemphigus. A negative result of direct immunofluorescence was observed in 3 patients, with two of them being elderly men (mean age: 71 years) and one with mucosal lesions only. In a study conducted by Alexandru et al. [45] the sensitivity of the direct immunofluorescence of anagen plucked hair equaled 100% (12/12 patients with pemphigus vulgaris). In this study 4 out 12 patients had no scalp involvement and one patient was in clinical remission [45]. Daneshpazhooh et al. suggested that the direct immunofluorescence of plucked hairs is a suitable alternative to the traditional DIF of the skin or mucous membranes [43]. The value of the direct immunofluorescence of telogen hairs remains controversial. In the study by Alexandru et al. [45] the direct immunofluorescence of plucked telogen hairs from 3 patients with pemphigus vulgaris was negative, whereas Tanasilovic et al. [46] reported a positive immunofluorescence of outer root sheath in both anagen and telogen hairs of all patients with active pemphigus vulgaris (27/27) and pemphigus foliaceus (8/8), irrespective of scalp involvement. The authors reported that the direct immunofluorescence of plucked telogen hairs was negative in all patients with pemphigus vulgaris or pemphigus foliaceus in remission [46]. According to European guidelines on pemphigus management, plucked hairs may be utilized for DIF for the diagnosis of pemphigus in specialized laboratories [47].

Rao et al. reported on the applicability of the direct immunofluorescence of plucked hairs in monitoring pemphigus activity [48]. The authors observed a positive direct immunofluorescence of plucked hair in a patient with a clinical remission and a negative result of direct immunofluorescence of the skin, suggesting that plucked hair is even a more reliable substrate for direct immunofluorescence than the skin [48]. However, the study was performed on a total of 5 patients with pemphigus vulgaris, no anti-desmoglein enzyme-linked immunoassay was performed, and indirect immunofluorescence was performed on two fixed serum dilutions, which constitute the limitations of the study [48]. A negative direct immunofluorescence of plucked hairs was reported to be a marker of remission [7]. In a recent study of Rai and Harikumar the usability of hair DIF in the assessment of immunological remission was evaluated in a total of 30 patients with pemphigus vulgaris in clinical remission [49]. The clinical remission was defined as no new/nonhealing lesions for at least 6 months in patients being off therapy or being treated with maximum dose of 10 mg of prednisolone daily with or without immunosuppressive therapy such as azathioprine in the dose of 50 mg daily or cyclophosphamide in the dose of 50 mg daily [49]. Positive hair DIF was observed in 14 patients, whereas positive DIF of the skin was present in 10 patients [49]. In 6 patients, hair DIF was positive, while DIF of the skin was negative, which may indicate that hair DIF is a better tool in evaluating immunological remission in patients with pemphigus vulgaris [49]. Daneshpazhooh et al. suggest repeating the DIF of plucked hairs in patients in clinical remission until the negativization and afterwards, performing a conventional DIF [7].

### 3.6. Trichoscopy of Pemphigus Lesions

Three full-text articles about trichoscopy (dermoscopy of the scalp and hairs) were identified.

Firstly, Pirmez published a case report which described the presence of acantholytic casts as a trichoscopic feature of pemphigus vulgaris. The author regarded this feature as Nikolsky's sign of the scalp indicating the activity of the disease [50].

In 2014, we described the trichoscopic features of scalp involvement in 9 patients with pemphigus vulgaris and 10 patients with pemphigus foliaceus [51]. The most common trichoscopic features of pemphigus were extravasations occurring in 95% (18/19) of patients with pemphigus and yellow haemorrhagic crusts present in 58% (11/19) of all patients. White polygonal structures were characteristic of pemphigus foliaceus and were visualized in 60% (6/10) of patients with pemphigus foliaceus, whereas vascular abnormalities were more common in pemphigus vulgaris and correlated with the severity of pemphigus measured with scalp PDAI.

In 2017, our group described trichoscopic features of 26 patients with pemphigus vulgaris and 17 patients with pemphigus foliaceus [52]. We concluded that the presence of extravasations (20/26, 76.9%), yellow haemorrhagic crusts (19/26, 73.1%), dotted vessels with whitish halo (6/26, 23.1%), circular vessels (6/26, 23.1%), or polymorphic vessels (6/26, 23.1%) can suggest a diagnosis of pemphigus vulgaris [52]. The presence of dotted vessels, dotted vessels with whitish halo, clustered dotted vessels, dotted vessels forming “string of pearls,” and lace-like vessels correlated positively with PDAI scalp activity score [52]. The combination of extravasations (12/17, 70.6%), yellow haemorrhagic crusts (12/17, 70.6%), yellow diffuse scaling (9/17, 52.9%), and tubular scaling (7/17, 41.2%) can indicate a diagnosis of pemphigus foliaceus [52].

### 3.7. Response to the Treatment

Cases of treatment-resistant scalp lesions in the course of pemphigus were reported [19, 24, 28–32, 53]. Kamphausen et al. [53] reported a case of a 48-year-old man with a severe involvement of the scalp with erosions, crusting, vegetative granulations, and alopecia. The patient was treated with protein A immunoadsorption, intravenous rituximab, intravenous dexamethasone pulses, and oral azathioprine. Complete healing of lesions was observed after 12 weeks [53]. Mlynek et al. reported a case of pemphigus foliaceus not responding to oral prednisolone at the dose of 1 mg/kg daily, with the intolerance of azathioprine, manifesting as a significant elevation of liver enzymes. The patient was treated with oral prednisolone at a dose of 1 mg/kg daily combined with intravenous immunoglobulins at a dose of 2 g/kg over 5 days in two cycles given at a 4-week interval, followed by eight immunoaphereses. After a 5-month follow-up the healing of scalp lesions with complete hair regrowth was observed. However, new lesions were still, although sporadically, observed [25].

Contrary to the cases above, Tyros et al. reported a successful treatment of pemphigus foliaceus localized on the scalp and the nose in a patient treated with pimecrolimus cream once daily for 4 weeks as monotherapy [22]. Good response to another topical calcineurin inhibitor, tacrolimus, as monotherapy was observed by Termeer et al. [23].

In our recent paper, we described the efficacy of perilesional and intralesional triamcinolone acetonide injections in two patients with residual scalp involvement in the course of pemphigus vulgaris [54]. Complete clearance of scalp lesions was achieved after, respectively, one and two perilesional and intralesional triamcinolone acetonide (10 mg/ml) injections, showing its possible usability in the treatment of recalcitrant scalp lesions in pemphigus [54].

In conclusion, to date there have not been sufficient convincing data to prove that scalp involvement in pemphigus is associated with poor response to treatment.

Detailed literature data on scalp involvement in pemphigus are shown in Table S1.

## 4. Conclusions

The scalp is a common location for pemphigus because of a high load of pemphigus antigens distributed in hair follicles. A high concentration of pemphigus antigens in hair follicles makes them a perfect substrate for direct immunofluorescence. Another useful diagnostic method is trichoscopy, especially in cases of pemphigus lesions limited to the scalp. The evaluation of response to treatment of patients with a severe scalp involvement requires large prospective observational studies.

## Figures and Tables

**Table 1:** Pemphigus Disease Area Index (PDAI) for the scalp.

Scalp	
Erosion/blisters or new erythema	Postinflammatory hyperpigmentation or erythema from resolving lesion
0—absent	0—absent
1—one quadrant	1—present
2—two quadrants 3—three quadrants 4—affecting the whole skull 10—at least one lesion > 6 cm	

## Supplementary Materials

Supplementary Materials Table S1: literature data on scalp involvement in pemphigus.
