# Supplementary material for: The Significance of Scalp Involvement in Pemphigus: A Literature Review
Source: Biomed Res Int. 2018 Mar 25;2018:6154397. doi: 10.1155/2018/6154397 (PMC5889856; doi:10.1155/2018/6154397)
Supplement: Supplementary Materials — Table S1: literature data on scalp involvement in pemphigus. [file 6154397.f1.docx]

| Table S1. Literature data on scalp involvement in pemphigus. | | | | | | | | | | | | | | | | | | | | |
| --- | --- | --- | --- | --- | --- | --- | --- | --- | --- | --- | --- | --- | --- | --- | --- | --- | --- | --- | --- | --- |
| **First author**  **+Year** | **Type**  **of article** | **PV^a^/**  **PF^b^** | **Sex/**  **Age [years]** | **Duration**  **of the scalp**  **involve-ment** | **Pull test** | **‘Normal anagen effluvium’** | | **Histo-**  **pathology** | **Scalp/Hair DIF^c^** | **IIF^d^** | **Anti-Dsg1^e^ antibody concen-tration (U/ml, ELISA^f^)** | | | **Anti-Dsg3 ^g^**  **antibody concen-tration (U/ml, ELISA)** | | ***Swab cultures/***  **trichoscopic**  **features** | **Treatment** | **Scalp lesions resistant to treatment** | | **Relapsing**  **scalp**  **lesions** |
| **Non-scarring alopecia** | | | | | | | | | | | | | | | | | | | | |
| **Veraitch,2013 [1]** | Original article (5 patients) | PV | F^h^/39 | 153 months (range 16-380 months) | + | + | | suprabasilar acantholysis within the ORS^i^, superficial cell infiltration consisting of lymphocytes and neutrophils, not observed in the lowermost bulbar region of the hair follicle, hair follicle density and phase were not affected | Scalp DIF - intercellular staining in the ORS | n.a.^j^ | 89 | | 150 | | *Staphylococcus epidermidis* | | Systemic corticosteroid and azathioprine, detailed data not available | | - | + |
|  |  |  | F/49 |  | + | + | |  | Scalp DIF - intercellular staining in the ORS | n.a. | 117 | | 148 | | n.a. | | Systemic corticosteroid and azathioprine, detailed data not available | | - | + |
|  |  |  | F/43 |  | + | + | |  | Scalp DIF - intercellular staining in the ORS | n.a. | 118 | | 213 | | *Staphylococcus aureus* | | Systemic corticosteroid and azathioprine, detailed data not available | | - | + |
|  |  |  | M^k^/32 |  | + | + | |  | Scalp DIF - intercellular staining in the ORS | n.a. | 69 | | 150 | | *Klebsiella pneumoniae* | | Systemic corticosteroid, cyclosporine, IVIg^l^, azathioprine, detailed data not available | | - | + |
|  |  |  | F/69 |  | + | + | |  | Scalp DIF - intercellular staining in the ORS | n.a. | 0 | | 206 | | n.a. | | Systemic corticosteroid, detailed data not available | | - | - |
| **Hadayer, 2013 [2]** | Case report | PV | F/32 | 2 years | n.a. | n.a. | | Acantholysis of the epidermis and ORS | Scalp DIF -  IgG deposits in intercellular spaces within the epidermis | n.a. | n.a. | | n.a. | | *Staphylococcus aureus* | | Systemic corticosteroid, mycophe-nolate mofetil, high potency topical corticosteroid  detailed data not available | | + | - |
| **Mlynek, 2009 [3]** | Case report | PF | F/14 | 2 months | n.a. | n.a. | | subcorneal acantholysis including the infundibular epithelium | Scalp DIF: infundibular and infra-infundibular intercellular IgG^m^ deposition | 1:2560 * | 2027 U/ml | | negative | | n.a. | | Oral prednisolone (1 mg/kg/day), two cycles of IVIg 2 g/kg over 5 days at a 4-week interval | | - | - |
| **Scarring alopecia** | | | | | | | | | | | | | | | | | | | | |
| **Gaitanis, 2013 [4]** | Case report | PV | F/64 | 6 years | + | n.a. | Suprabasal clefting involving the hair and the adjacent epidermis,  disintegration of underlying pilosebaceous units, follicular acantholysis | | n.a. | n.a | n.a | n.a | | | Negative | | Oral methyl-prednisolone 64 mg/day, mycopheno-late mofetil  4 g/day, topical mometasone furoate | - | | - |
| **Ko,**  **2011 [5]** | Case report | PV | M/51 | 20 years | n.a. | n.a. | Acantholysis with the formation of intra-epidermal blisters and the separation of the epidermis, clustering of several adjacent hair follicles with perifollicular inflammatory cell infiltration, a perivascular infiltrate with numerous lymphocytes and few eosinophils in the dermis | | Scalp DIF: positive C3 staining on the cell surface of keratinocytes | n.a. | n.a. | n.a. | | | *Staphylococcus epidermidis* | | Oral methyl-prednisolone, local disinfectant,  detailed data not available | + | | - |
| **Jappe, 2003 [6]** | Case report | PV | M/57 | 15 years | n.a. | n.a. | Acantholysis and blister formation in a predominantly suprabasal location, sometimes higher in the stratum spinosum of the follicular epithelium, foci of fibrosis in the upper dermis, the structure of hair papillae was unaffected | | Scalp DIF -intercellular deposits of immunoglobulins and complement within the epidermis | ME^n^-1:320 | Posi-tive | positive | | | *Staphylococcus aureus* | | Methyl-prednisolone  60 mg/day, mycophenolate mofetil 2 g/day | + | | - |
| **Petronić-Rosić, 1999 [7]** | Case report | PV | M/39 | 10 years | n.a. | n.a. | Several discrete hair follicles rooted in the subcutaneous fat anastomosing in the upper part of the dermis, sharing a common, dilated infundibulum, the upper part of the dermis with fibroplasia and a sparse perivascular lymphocytic infiltrate | | n.a. | n.a. | n.a. | n.a. | | | *Staphylococcus aureus* | | Oral prednisone, azathioprine, methotrexate, cyclophosphamide, systemic and topical antibiotics  detailed data not available | + | | - |
| **Saijyo, 1998 [8]** | Case report | PV | M/35 | 1 year | n.a. | n.a. | Intra-epidermal, focally suprabasal, acantholytic cleft formation, cellular infiltration in the papillary dermis, numerous plasma cells beneath the epidermis, lymphocytes and histiocytes in the edematous papillary dermis | | n.a. | 1:128 * (error in the titer in the original publication) | n.a. | n.a. | | | *Staphylococcus aureus* | | Oral prednisone 5 mg/every other day, triamcinolone acetonide injections | + | | - |
| **Pemphigus vegetans** | | | | | | | | | | | | | | | | | | | | |
| **Mori, 2014 [9]** | Case report | PV, vege-tans | M/42 | 2 years | n.a. | n.a. | Diffuse intra-epidermal cleavage in the basal layer of the epidermis,  an exudative and productive angioplastic lichenoid inflammation of the superficial dermis | | Scalp DIF - epidermal intercellular deposits of IgG | ME-1:160 | n.a. | n.a. | | | n.a. | | Oral prednisone 1 mg/kg/day | - | | - |
| **Lehrhoff, 2012 [10]** | Case report | PV, vege-tans | M/58 | 8 months | n.a. | n.a. | Intraepidermal blister with suprabasal acantholysis, superficial, perivascular infiltrate with  eosinophils | | n.a. | ME-1:320 | 90 U/ml | 34 U/ml | | | n.a. | | Oral prednisone 60 mg/day, methotrexate 7.5 mg/week, topical mometasone, intralesional gluco-  corticoid injections | n.a. | | - |
| **Kampf-hausen, 2012 [11]** | Corre-spon-dence | PV, vege-tans | M/48 | 12 | n.a | n.a | Consistent with PV (detailed data not available) | | Consistent with PV  (detailed data not available) | Consi-stent with PV (de-  tailed data not avail-able) | - | 388 U/ml | | | n.a. | | Immuno-adsorption (3 cycles), rituximab (1 g, once), dexametha-sone pulses (16 pulse), azathioprine (2.5 mg/kg/day)🡪mycopheno-late mofetil,  Treatment of relapse: rituximab (2 courses, with a 2-week interval), dexametha-sone (1 cycle), mycopheno-late mofetil | + | | + |
| **Dano-poulou, 2006 [12]** | Case report | PV, vege-tans | F/54 | 4 months | n.a. | n.a. | Papillomatous, proliferating, acantholytic lesions in the epidermis, eosinophilic infiltrate in the dermis | | Scalp DIF: intercellular IgG and C3 deposits mainly in the lower part of the epidermis | n.a. | Nega  tive | 158 | | | Staphylococcus epidermidis | | Oral prednisolone  60 mg/day, topical clobetasol propionate twice daily | - | | + |
| **Rackett, 1995 [13]** | Case report | PV, vege-tans | M/46 | n.a. | n.a. | n.a. | Indicative of pemphigus vegetans | | n.a | n.a. | n.a. | n.a. | | | Negative | | Oral prednisone 60 mg/day, azathioprine 100 mg/day, intralesional corticosteroid injections, dapsone 100 mg/day, intravenous methylprednisolone 1 g/day in a 5-day course, methotrexate 20 mg /week | + | | - |
| **Normal anagen effluvium** | | | | | | | | | | | | | | | | | | | | |
| **Fard, 2017 [14]** | Correspondence | 52 PV | 33F, 19M; average age 43.0±  16.8 | n.a. | + | 22/52 (42%) | n.a. | | n.a. | n.a. | 186.9  ±85.9 in pa-  tients with posi-  tive anagen pull test; 89.1  ±  100.1 in pa-  tients with nega-  tive anagen pull test;  p  <0.001 | 219.0  ±109.8 in pa-  tients with positive anagen pull test; 256.4  ±133.9 in patients with negative pull test  p=0.344 | | | n.a. | | n.a. | n.a. | | n.a. |
| **Daneshpazhooh, 2015 [15]** | Original article  (96 PV patients, including 57 patients with scalp involve-ment) | 57 PV | n.a. | n.a. | + | +  52/57 (91.2%) | n.a. | | n.a. | n.a. | n.a. | n.a. | | | n.a. | | n.a. | n.a. | | - |
| **Delmonte, 2000 [16]** | Corre-spon-dence | 3 PV | M/70 | 1 month | + | + | n.a. | | n.a. | ME 1:40 | n.a. | n.a. | | | n.a. | | Oral prednisone 1 mg/kg/day | - | | - |
| **Isolated scalp involvement** | | | | | | | | | | | | | | | | | | | | |
| **Tyros, 2013 [17]** | Case report | PF | M/79 | n.a. | n.a. | n.a. | Superficial bullae with acantholytic cells, parakeratosis, acanthosis and slight spongiosis | | n.a. | n.a. | n.a. | n.a. | | | n.a. | | 1% pimecrolimus cream once daily | - | | - |
| **Oretti, 2011 [18]** | Case report | PV | M/69 | 4 weeks  (relapse) | n.a. | n.a | Isolated  basal keratinocytes in a ‘tombstone’  fashion, suprabasal cleavage of the epidermis, acantholytic cells, follicular acantholysis of the ORS | | n.a. | 1:80 (substrate not mentioned) | n.a. | n.a. | | | n.a | | Oral methyl-prednisolone  1 mg/kg/day, oral azathioprine 150 mg/day | + | | - |
| **Ferrara, 2009 [19]** | Case report | PV | F/57 | 3 months | n.a. | n.a. | Suprabasal clefts with acantholytic keratinocytes | | Hair DIF -intercellular IgG deposits within the ORS | n.a. | n.a. | n.a. | | | n.a. | | Oral deflazacort 90 mg/day | - | | - |
| **Termeer, 2004 [20]** | Letter to the editor | PF | M/83 | 9 months | n.a. | n.a. | Split in the upper granular layer of the epidermis, a superficial bulla filled with scattered acantholytic keratinocytes and fibrin | | Scalp DIF -  Intercellular IgG deposition in the upper epidermal layers | n.a. | n.a. | n.a. | | | n.a. | | 0.1% tacrolimus ointment twice daily | - | | - |
| **Lapiere, 2004 [21]** | Letter | PV | F/66 | 7 years | n.a. | n.a. | Acantholysis with the formation of intraepidermal blisters above the basal layer, dense perivascular infiltrate with lymphocytes and eosinophils | | Scalp DIF -positive for IgG, IgA and C3 on the cell surface of keratinocytes | ME 1:320 | n.a. | n.a | | | *Staphylococcus aureus* | | Beta-methasone and fusidic acid | - | | - |
| **Trichoscopy** | | | | | | | | | | | | | | | | | | | | |
| **Sar-Pomian, 2017[22]** | Original article (68 patients) | 26 PV | n.a. | n.a. | n.a. | n.a. | n.a. | | n.a. | n.a. | n.a. | n.a. | | | Extravasations, yellow hemorrhagic crusts, dotted vessels with whitish hallo, circular vessels, polymorphic vessels‡ | | n.a. | n.a. | | n.a. |
|  |  | 17 PF | n.a. | n.a. | n.a. | n.a. | n.a. | | n.a. | n.a. | n.a. | n.a. | | | Extravasations, yellow hemorrhagic crusts, yellow diffuse scaling, tubular scaling‡ | | n.a. | n.a. | | n.a. |
| **Sar-Pomian, 2014 [23]** | Original article  (19 patients) | 9 PV | 4F, 5M, avera-ge age:  58.8+/-19.7 years | Average:  2.1+/-2.4 years | n.a. | n.a. | n.a. | | n.a. | n.a. | n.a. | n.a. | | | Extravasations, linear serpentine vessels, yellow dots, yellow hemorrhagic crusts¶ | | n.a. | n.a. | | n.a. |
|  |  | 10 PF | 3F, 7M  Aver-age age: 64.2+/-16.4 years | Average: 2.9+/-4.0 years | n.a. | n.a. | n.a. | | n.a. | n.a. | n.a. | n.a. | | | White diffuse scaling, extravasations, yellow hemorrhagic crusts, white polygonal structures¶ | | n.a | n.a. | | n.a |
| **Pirmez, 2012 [24]** | Case report | PV | F/57 | n.a. | + | + | n.a. | | n.a. | n.a. | n.a. | n.a. | | | n.a. | | n.a. | n.a. | | - |

^a^PV - pemphigus vulgaris, ^b^PF - pemphigus foliaceus ^c^DIF - direct immunofluorescence, ^d^IIF - indirect immunofluorescence, ^e^ anti-Dsg1 - anti-desmoglein 1 antibody, ^f^ ELISA - enzyme-linked immunoassay, ^g^ anti-Dsg3 - anti-desmoglein 3 antibody, ^h^ F - female, ^i^ ORS - outer root sheath, ^j^ n.a. - not addressed, ^k^ M - male,^l^ IVIg - intravenous immunoglobulin, ^m^ IgG - immunoglobulin G, ^h^ ME - monkey esophagus , * indirect immunofluorescence substrate not reported, ‡trichoscopic features suggesting a diagnosis, ¶ - trichoscopic features occurring in more than 50% of patients

1. Veraitch O, Ohyama M, Yamagami J, Amagai M. Alopecia as a rare but distinct manifestation of pemphigus vulgaris. *J Eur Acad Dermatol Venereol.* 2013; 27: 86-91

2. Hadayer N, Ramot Y, Maly A, Zlotogorski A. Pemphigus vulgaris with loss of hair on the scalp. *Int J Trichology.* 2013; 5: 157-8

3. Mlynek A, Bar M, Bauer A, Meurer M. Juvenile pemphigus foliaceus associated with severe nonscarring alopecia. *Br J Dermatol.* 2009; 161: 472-4

4. Gaitanis G, Patmanidis K, Skandalis K, Alexis I, Zioga A, Bassukas ID. Scaring alopecia in pemphigus vulgaris: a rare or underdiagnosed presentation? *Eur J Dermatol.* 2013; 23: 253-5

5. Ko DK, Chae IS, Chung KH, Park JS, Chung H. Persistent pemphigus vulgaris showing features of tufted hair folliculitis. *Ann Dermatol.* 2011; 23: 523-5

6. Jappe U, Schroder K, Zillikens D, Petzoldt D. Tufted hair folliculitis associated with pemphigus vulgaris. *J Eur Acad Dermatol Venereol.* 2003; 17: 223-6

7. Petronic-Rosic V, Krunic A, Mijuskovic M, Vesic S. Tufted hair folliculitis: a pattern of scarring alopecia? *J Am Acad Dermatol.* 1999; 41: 112-4

8. Saijyo S, Tagami H. Tufted hair folliculitis developing in a recalcitrant lesion of pemphigus vulgaris. *J Am Acad Dermatol.* 1998; 38: 857-9

9. Mori M, Mariotti G, Grandi V, Gunnella S, Maio V. "Pemphigus vegetans of the scalp". *J Eur Acad Dermatol Venereol.* 2016; 30: 368-70

10. Lehrhoff S, Miller K, Fischer M, Kamino H, Meehan S. Localized pemphigus with vegetative features. *Dermatol Online J.* 2012; 18: 11

11. Kamphausen I, Schulze F, Schmidt E, Zillikens D, Kunz M. Treatment of severe pemphigus vulgaris of the scalp with adjuvant rituximab and immunoadsorption. *Eur J Dermatol.* 2012; 22: 786-7

12. Danopoulou I, Stavropoulos P, Stratigos A, Chatziolou E, Chiou A, Georgala S, et al. Pemphigus vegetans confined to the scalp. *Int J Dermatol.* 2006; 45: 1008-9

13. Rackett SC, Rothe MJ, Hoss DM, Grin-Jorgensen CM, Grant-Kels JM. Treatment-resistant pemphigus vegetans of the scalp. *Int J Dermatol.* 1995; 34: 865-6

14. Fard GD, Khosravi H, Ghayoumi A, Balighi K, Ghandi N, Teimourpour A, et al. Anagen hair loss, anti-desmoglein 1, and pemphigus disease area index: a significant relationship? *J Dtsch Dermatol Ges.* 2017; 15: 946-8

15. Daneshpazhooh M, Mahmoudi HR, Rezakhani S, Valikhani M, Naraghi ZS, Mohammadi Y, et al. Loss of normal anagen hair in pemphigus vulgaris. *Clin Exp Dermatol.* 2015; 40: 485-8

16. Delmonte S, Semino MT, Parodi A, Rebora A. Normal anagen effluvium: a sign of pemphigus vulgaris. *Br J Dermatol.* 2000; 142: 1244-5

17. Tyros G, Kalapothakou K, Christofidou E, Kanelleas A, Stavropoulos PG. Successful treatment of localized pemphigus foliaceus with topical pimecrolimus. *Case Rep Dermatol Med.* 2013; 2013: 489618

18. Oretti G, Giordano D, Di Lella F, Gradoni P, Zendri E, Ferri T. Unilesional pemphigus vulgaris of the scalp after cochlear implantation. *Am J Otolaryngol.* 2011; 32: 80-1

19. Ferrara G, Massone C, Zalaudek I, Argenziano G. Unilesional pemphigus vulgaris of the scalp. *Dermatol Online J.* 2009; 15: 9

20. Termeer CC, Technau K, Augustin M, Simon JC. Topical tacrolimus (protopic) for the treatment of a localized pemphigus foliaceus. *J Eur Acad Dermatol Venereol.* 2004; 18: 636-7

21. Lapiere K, Caers S, Lambert J. A case of long-lasting localized pemphigus vulgaris of the scalp. *Dermatology.* 2004; 209: 162-3

22. Sar-Pomian M, Rudnicka L, Olszewska M. Trichoscopy - a useful tool in the preliminary differential diagnosis of autoimmune bullous diseases. *Int J Dermatol.* 2017; 56: 996-1002

23. Sar-Pomian M, Kurzeja M, Rudnicka L, Olszewska M. The value of trichoscopy in the differential diagnosis of scalp lesions in pemphigus vulgaris and pemphigus foliaceus. *An Bras Dermatol.* 2014; 89: 1007-12

24. Pirmez R. Acantholytic hair casts: a dermoscopic sign of pemphigus vulgaris of the scalp. *Int J Trichology.* 2012; 4: 172-3
